# Dysmenorrhœa

**Published:** 1871-12

**Authors:** 


Dysmenorrhœa.—Dr. W. K. Boling (*Nashville Jour. of Medicine and Surgery*) reports the case of a patient who had suffered from dysmenorrhœa, who had never been pregnant, and who had never suffered in her turns before marriage. A pil. of opium, camphor, and lupulin was prescribed, with the effect of quieting the pain, but the after effect of the opium being disagreeable, she could not be again induced to resort to opium. She continued to suffer at each period for several months. She then, at the beginning of a pain, sat a few moments on a chamber in which a drachm of spirits of ammonia had been placed. In a minute the pain was gone, and did not return. The same result follows at each period since.

